# Synthetic Biology‐Based Engineering Living Therapeutics for Antimicrobial Application

**DOI:** 10.1002/EXP.20240045

**Published:** 2025-04-03

**Authors:** Shun Huang, Shuihao Zhao, Haijie Zhao, Mingzhang Wen, Zhong Guo

**Affiliations:** ^1^ Key Laboratory of Cell Proliferation and Regulation Biology of Ministry of Education Guangdong Zhuhai‐Macao Joint Biotech Laboratory Faculty of Arts and Sciences Beijing Normal University Zhuhai China; ^2^ Department of Pharmaceutical Sciences University of Basel Basel Switzerland; ^3^ Frontiers Science Center for Synthetic Biology (Ministry of Education) Tianjin University Tianjin China; ^4^ Zhejiang Institute of Tianjin University Shaoxing China

**Keywords:** antimicrobial therapy, engineering living therapeutics, synthetic biology

## Abstract

There is currently a pressing issue of antimicrobial resistance, with numerous pathogenic superbugs continually emerging, posing significant threats to both human health and the economy. However, the development of new antibiotics has not kept up in pace with the development of microbial resistance, necessitating the exploration of more effective approaches to combat microbes. Synthetic biology offers a novel paradigm by employing selective screening and assembling diverse biological components to redesign biological systems that can specifically target and eliminate microbes. In particular, engineering living therapeutics enables the detection and precise eradication of pathogenic microorganisms in a controlled means. This review provides an overview of recent advancements in engineering living therapeutics using synthetic biology for antibacterial treatment. It focuses on modifying bacteriophages, microbes, and mammalian cells through engineering approaches for antibacterial therapy. The advantages of each approach are delineated along with potential challenges they may encounter. Finally, a prospective outlook is presented highlighting the potential impact and future prospects of this innovative antimicrobial strategy.

## Graphical abstract

This perspective highlights synthetic biology‐driven antibacterial strategies, focusing on three innovative approaches: engineered bacteriophages for precision bacterial targeting, reprogrammed microbes that detect quorum‐sensing signals or metabolites to release antimicrobials, and engineering mammalian cells that recognize pathogen‐associated molecular patterns to initiate antibacterial responses. These approaches leverage genetic circuits to combat antibiotic resistance with high specificity, offering innovative solutions beyond traditional antibiotics and addressing multidrug‐resistant infections.

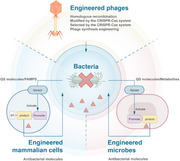

## 1 Introduction

Microorganism constitute one of the most abundant species on Earth, and are intricately linked to numerous diseases in humans. Following Alexander Fleming's discovery of penicillin in 1928, significant advancements were made in the effective treatment of microbial infections [1]. A survey indicated that the use of antibiotics has significantly increased human life expectancy [2]. However, as antibiotics have become extensively utilized globally, microbes have also evolved to develop corresponding drug resistance, presenting a new challenge for antimicrobial therapy. Antimicrobial resistance (AMR) has emerged as one of the most urgent issues in global public health crises, particularly concerning a group of bacteria known as ESKAPE that are both highly lethal and resistant [3]. Currently, drug‐resistant bacterial infections are responsible for over 700,000 deaths each year worldwide. A report revealed that in 2019 alone, there were 4.95 million deaths associated with AMR [4]. According to estimates from the World Health Organization (WHO), by 2050, it is projected that the mortality toll will surpass 10 million people, resulting in a staggering economic loss of one trillion USD per year [5]. This phenomenon represents one of the gravest threats to human, animal, agricultural, and environmental well‐being [6].

In light of the threat of drug‐resistant bacteria, the WHO is urging researchers to design innovative antibacterial approaches for combating critical antibiotic‐resistant infections [7]. While new antibiotic development is undoubtedly the most direct avenue, it fails to keep up with the increasing rate of bacterial resistance [5]. Furthermore, regulatory and marketing procedures have slowed down the progress in creating innovative antibiotics [8]. Additionally, using antibiotics can lead to the emergence of new resistance mechanisms. Therefore, it is necessary to explore alternative therapeutic strategies such as combination therapy, high‐molecular‐weight biomaterials, nanotechnology, and antibacterial phytochemicals [9]. Synthetic biology also offers a promising avenue for advancing antimicrobial therapy. This cutting‐edge research domain harnesses engineering principles to construct living systems from biological components, aiming to imbue them with specific biological functions. Genetic circuits and systems can be encoded in particular organisms to manipulate cellular metabolism in response to specific signals, ultimately achieving the desired functionalities [10]. Over recent decades, synthetic biology has undergone rapid development. As early as the beginning of the 21st century, researchers initiated studies on designing genetic circuits and switches [11]. With the discovery of an increasing number of biological components [12], particularly with the emergence of gene editing technologies such as the CRISPR‐Cas system [13] and the development of high‐throughput sequencing methods [14], increasingly intricate and robust genetic circuits have been designed. These circuits have found diverse applications, such as the creation of CAR‐T cell therapy by modifying T cells for the treatment of B‐cell acute lymphoblastic leukemia, which has been clinically applied and commercialized [15]. Additionally, synthetic biology enables the production of drugs like artemisinin through microbial fermentation for disease treatment [16]. Moreover, engineered microorganisms hold promise for disease detection, such as diabetes [17]. These developments establish a novel therapeutic approach using living organisms, providing a new mindset and significant potential for utilizing synthetic biology to engineer living organisms for antimicrobial therapy.

In this review, we delve into the utilization of synthetic biology techniques for designing a unique antibacterial system. We compile and present the latest advancements in this area, examine a new antibacterial strategy through the engineering living therapeutics like bacteriophages, microbes, and mammalian cells, and evaluate the advantages and limitations of each approach. Additionally, we provide insights into the future prospects of synthetic biology technologies in antibacterial research and their potential application in treating other diseases.

## 2 Engineering Phages

Bacteriophages are viruses that infect bacteria. They have coevolved with bacteria for approximately four billion years and their population is estimated to be on the order of 10^31^, which is greater than the combined total of other organisms [18]. In 1917, Felix D'Herelle made a significant discovery of bacteriophages [19], and in 1919, he successfully used them for the first time to treat bacterial dysentery in children. From then until the 1940s, bacteriophages were extensively utilized for treating bacterial infections in humans and animals [20]. However, with the introduction of penicillin in the 1940s, most countries shifted their focus toward antibiotics except Eastern Europe and the Soviet Union where research on phage therapy continued [21].

Due to the rise of drug‐resistant bacteria, especially strains that are resistant to multiple drugs phage therapy has gained renewed attention as an alternative to antibiotics [22]. For example, a report from 2020 outlined the treatment of 13 patients severely infected with *Staphylococcus aureus* (*S. aureus*) using three bacteriophages from the *Myoviridae* family that were administered intravenously [23]. Another report in 2022 involved the screening and modification of two phages, which were co‐injected into a male patient suffering from cystic fibrosis and refractory *Mycobacterium abscessus* (*M. abscessus*) infection. This led to an improvement in the lung infection, ultimately resulting in the successful performance of lung transplantation [24]. However, phage therapy still faces several challenges, such as difficulty in isolating naturally occurring phages [25], limited host ranges, and low infection efficiency [26]. Engineering and synthesizing bacteriophages using genetic engineering methods can address these challenges by improving phage production rates for easier acquisition, expanding the host range of bacteriophages, and increasing infection efficiency. Typically, two strategies are employed for phage engineering: genetic modification of known phages (Table 1), and synthesis/construction of phage genes based on genomics [27].

**TABLE 1:** Genetic modification of phages and antibacterial applications.

Methods	Phages	Purposes	Applications and results
Homologous recombination	T5	Modify tail fiber protein to increase host range.	The engineered phages exhibited absorption rates ranging from 28.10% to 99.49%, compared to the original rates of 0.28% to 28.84%.
	T7	Modified T7 variant against pathogenic *E. coli* that causes urinary tract infections.	Effectively eliminating bacteria at low multiplicity of infection (MOI).
	K64‐1	The phage carrying capsule depolymerases was screened out, which increased the infection rate of *Klebsiella*.	Subsequent study has shown promising results in mouse models.
	ZoeJ	Effective lytic phages have been developed to treat mycobacterial infections.	After treatment, the patient continued to improve clinically, the surgical wound and skin lesions gradually healed, lung and liver function improved.
CRSIPR‐Cas9	Selz	Engineered phage display cell penetrating peptides (CPPs) can improve the killing of *Salmonella*.	Significantly killed *Salmonella* SL1344 intracellularly in Hela and A549 cells, reaching 64% and 48%, respectively.
	HEPTs	Heterologous antimicrobial genes are integrated into phages to eliminate *E. coli*.	It showed remarkable bactericidal effect in urinary tract infection model.
	P1	The antimicrobial activity of *S. flexneri* using the P1 phage‐enabled CRISPR‐Cas9 system.	The cleaning effect of *S. flexneri* infection was realized in zebrafish larva model.
	φ SaBov	Incorporating the CRISPR/Cas9 system into the bacteriophage genome and supplementing the tail fiber protein of the bacteriophage to enhance its lethality against *S. aureus*.	In the mouse in model, treatment of infected skin areas with φ SaBov‐Cas9‐nuc successfully reduced the number of surviving *S. aureus*.
CRSIPR‐Cas3	SNIPR001	Engineering of tail fiber proteins and CRISPR‐Cas systems for specific targeting of *E. coli*.	In a mouse colitis model, SNIPR001 led to a reduction of 4 log_10_ CFU g^−1^ in *E. coli* levels. Moreover, in a minipig model, it exhibited excellent tolerance and gastrointestinal recovery.
	ФCD24‐2	Using phage‐delivered CRISPR‐Cas3 antimicrobials to target *C. difficile*.	Effectively reduced the burden of *C. difficile* and clinical symptoms of disease in mice.

*Note: E. coli, Escherichia coli; S. flexneri, Shigella flexneri; S. aureus, Staphylococcus aureus; C. difficile, Clostridioides difficile*.

### 2.1 Homologous Recombination

**FIGURE 1:**
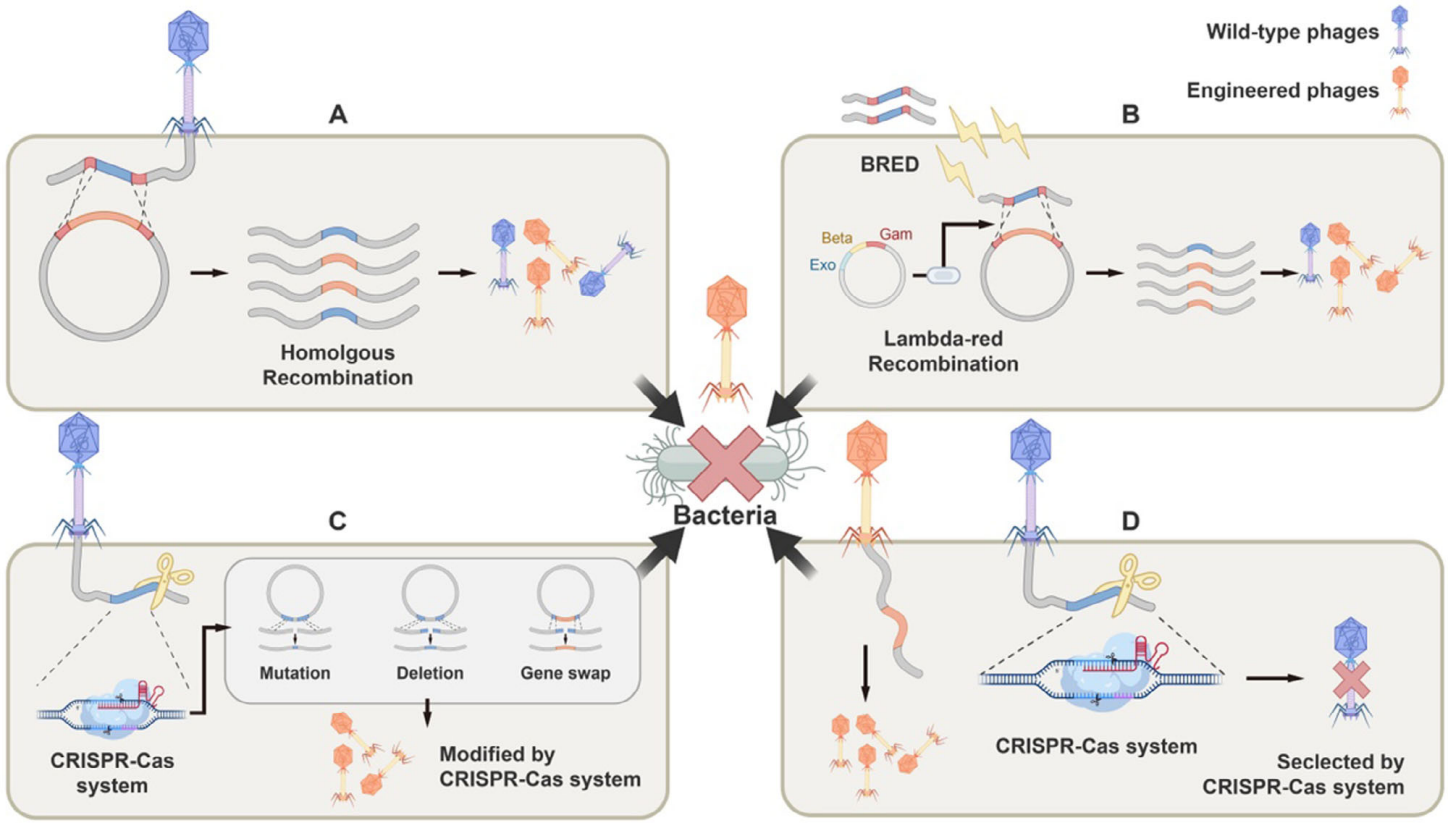
Modification and counter‐selection strategies for bacteriophage genome. (A) Modification of bacteriophage genome is achieved through homologous recombination. (B) BRED (bacteriophage recombineering of electroporated DNA) technique is employed to introduce bacteriophage DNA, template DNA, and the aforementioned recombinase system into host cells, thereby enhancing transformation efficiency. (C) Modification of bacteriophage genome is carried out utilizing the CRISPR‐Cas system. (D) The CRISPR‐Cas system is utilized for the selection of engineered bacteriophages.

Homologous recombination is a commonly employed method in genetic engineering [28]. Bacteriophage recombination, also known as phage hybridization, stands out as one of the earliest approaches for modifying bacteriophages [29] (Figure 1A). It is important to note that the rate of such recombination is generally modest, typically falling within the range of 10^−10^ to 10^−4^ [26].

The utilization of recombination enzymes can enhance the efficiency of homologous recombination. For instance, the lambda‐red recombination system, consisting of EXO, Beta, and Gam proteins, has demonstrated promising results when introduced into *Escherichia coli* (*E. coli*) [30]. Researchers have also exploited the property of recombination enzymes to modify bacteriophage genes in *Mycobacterium tuberculosis* (*M. tuberculosis*) [31], *Salmonella typhimurium* [32], and *Klebsiella pneumoniae* (*K. pneumoniae*) [33], showcasing increased efficacy. Additionally, the technique of bacteriophage recombineering of electroporated DNA (BRED) increases transformation rates by introducing bacteriophage DNA, template DNA, and the above‐mentioned recombinase system into host cells, particularly effective for Gram‐positive bacteria with thicker cell walls [31] (Figure 1B).

The aforementioned techniques effectively enable the directed mutagenesis of wild‐type bacteriophage genomes, addressing certain challenges associated with naturally occurring bacteriophages while opening avenues for new research and the development of novel therapeutic methods. For example, by employing homologous recombination, the substitution of the wild‐type T5 bacteriophage's long tail fiber protein (pb1) gene with a gene (BD13) encoding a protein with broad host recognition significantly broadens the host range [34]. In the case of the wild‐type T7 bacteriophage, researchers have made single amino acid substitutions in the bacteriophage's receptor binding protein (RBP), enhancing infectivity against bacteria and showing therapeutic potential in a urinary tract model of *E. coli* infection [35]. On the other hand, with the implementation of the recombination system and BRED technology, deletion mutants of bacteriophage K64‐1 have been constructed, allowing for the screening of bacteriophages carrying capsule depolymerases. This enhancement increases infection rates against *Klebsiella* and assists in combating multidrug‐resistant *Klebsiella* infections [33]. Subsequent investigations have utilized screened bacteriophages to treat *K. pneumoniae* infections in mice, resulting in improved survival rates observed after 30 days of treatment, providing comprehensive data and references for potential clinical applications [36]. Furthermore, bacteriophages engineered using BRED technology have progressed to clinical trials. The engineered ZoeJ phage was administered to treat a 15‐year‐old patient with cystic fibrosis and disseminated *M. abscessus* infection, showing promising results in clinical observation [37].

### 2.2 Phage Modification Based on the CRISPR‒Cas System

The CRISPR‐Cas system plays a crucial role in bacterial immune systems, defending against the intrusion of exogenous genes into cells, especially in defense against bacteriophage invasion. Its primary mechanism involves RNA‐guided cleavage of target DNA with remarkable specificity. With time, it has evolved into a mainstream genetic editing tool in contemporary research [38]. Leveraging this system, we can manipulate bacteriophage genomes, thereby modifying the characteristics of bacteriophages to achieve our desired state (Figure 1C). Additionally, it enables the selective removal of wild‐type bacteriophage genes through strategic design while retaining the modified ones (Figure 1D).

#### 2.2.1 Modification of Phage Genomes

The CRISPR‐Cas system comprises a broad classification, categorized into two classes, six types, and 33 subtypes, based on various combinations of Cas protein genes [39]. Among these, the CRISPR‐Cas9 system in class II‐A stands out as the most prevalent editing tool, frequently employed in both genome editing and bacteriophage modification. As early as 2014, researchers conducted a study on editing the genome of virulent bacteriophage 2972 using the naturally occurring Type II‐A class CRISPR‐Cas system of *Thermophilic streptococcus* DGCC7710. This facilitated selective mutation, deletion, and gene exchange within the bacteriophage's genome [40].

The CRISPR‐Cas9 system was employed to modify genes within the Ig‐like domain of bacteriophage Selz, enabling the binding of the GP94 short peptide with penetrating peptides. This enhancement augmented the bacteriophage's efficacy in infecting *Salmonella*, offering a potential new solution to address challenges posed by *Salmonella* infections and antibiotic resistance [41]. In another study, this system was employed to integrate heterologous antibacterial genes (such as colicin E7 from *E. coli*) into the bacteriophage genome. This modification endowed the engineered bacteriophage with the ability to accelerate bacterial lysis, effectively eradicating various pathogens in a urinary tract infection model [42]. Furthermore, integrating the CRISPR‐Cas9 system into the bacteriophage genome allows for its activation within infected host cells, leading to rapid bacterial death. This strategy was applied in P1 bacteriophages to combat dysentery caused by *Shigella flexneri* infection, demonstrating efficacy in a zebrafish larval model [43]. Notably, inducing mutations in the fiber protein at the bacteriophages tail end is essential to broaden their host range, a significant aspect in combating antibiotic‐resistant bacteria and exploring new antibacterial strategies [44]. The fusion of this engineering approach with the CRISPR‐Cas9 system yielded engineered bacteriophages φ SaBov and φ 11, which serve as potent antibacterial agents against *S. aureus*. Their therapeutic effectiveness was significantly improved in both in vitro and mouse models [45].

Within Type I CRISPR‐Cas systems, the CRISPR‐Cas3 system is a commonly utilized tool. Introducing the I‐E type CRISPR‐Cas system and a DNA template into *Vibrio cholerae* (*V. cholerae*), enabled precise gene deletion and insertion in phages [46]. One such bacteriophage, known as SNIPR001, when combined with the CRISPR‐Cas3 (I‐E type) system, accurately eliminates *E. coli*, a pathogen known to infect hematological cancer patients, while exhibiting good tolerance in mouse and minipig models [47]. In another study, the engineered bacteriophage ФCD24‐2 utilizes the CRISPR‐Cas3 (I‐B subtype) system to express a self‐targeting CRISPR. This guides the pathogen's endogenous CRISPR‐Cas3 to the bacterial genome, thereby enhancing the bacteriophage's ability to eliminate *Clostridium difficile* (*C. difficile*) by disrupting bacterial DNA [48]. Despite not being as straightforward as CRISPR‐Cas9 system currently, a recent study has demonstrated the superiority of CRISPR‐Cas3 in gene editing [49]. With ongoing technological advancements, it is believed that the CRISPR‐Cas3 system will find increasingly diverse applications in bacteriophage engineering.

#### 2.2.2 The Counterselection Strategy of the CRISPR‐Cas System

The counterselection strategy of the CRISPR‐Cas system plays a pivotal role in bacteriophage engineering and typically involves two distinct steps. Initially, bacteriophages are either constructed or modified through various techniques. However, even within the progeny of engineered bacteriophages, a large number of wild‐type bacteriophages may persist. Consequently, the subsequent step requires screening to obtain the desired progeny. Unlike bacteria, bacteriophage screening lacks reliance on resistance genes, rendering the process challenging. Although alternative markers have been reported for bacteriophage screening, their practical application remains limited [50]. However, the CRISPR‐Cas system offers a solution by selectively removing wild‐type bacteriophage genes while retaining engineered modifications. This reverse selection method significantly improves screening efficiency.

In a study, a counterselection strategy facilitated by the CRISPR‐Cas9 system effectively addressed the challenge of low recombination efficiency encountered in initial modification steps. Compared to solely utilizing single‐stranded BRED, the incorporation of the CRISPR system (CRISPR‐BRED) yielded nearly 100% modified progeny [51]. The method of CRISPR‐BRED described in the report also elucidated that recombination and counterselection can occur simultaneously [46], simplifying the engineering process. However, numerous bacteriophages have developed resistance mechanisms against the CRISPR‐Cas system, including alterations to DNA segments recognized by the inhibited Cas proteins [52]. Consequently, DNA‐targeting CRISPR‐Cas9 systems often exhibit robust immune evasion [53]. The Type VI (CRISPR‐Cas13) system, which targets RNA and can induce host cell dormancy, effectively reduces the generation of immune escape variants and is considered the optimal tool for screening recombinant bacteriophages [54]. Counterselection strategies mediated by CRISPR‐Cas13 have been demonstrated to have favorable outcomes in bacteriophage screening across *E. coli* [55] and *Pseudomonas aeruginosa* (*P. aeruginosa*) [56].

Undoubtedly, the screening process is an indispensable step in obtaining a large quantity of engineered bacteriophages. CRISPR‐Cas‐based screening has greatly simplified this process, thereby addressing the challenge of isolating natural bacteriophages. This development provides a significant foundation for the large‐scale production of bacteriophage therapies.

### 2.3 Phage Synthesis Engineering

**FIGURE 2:**
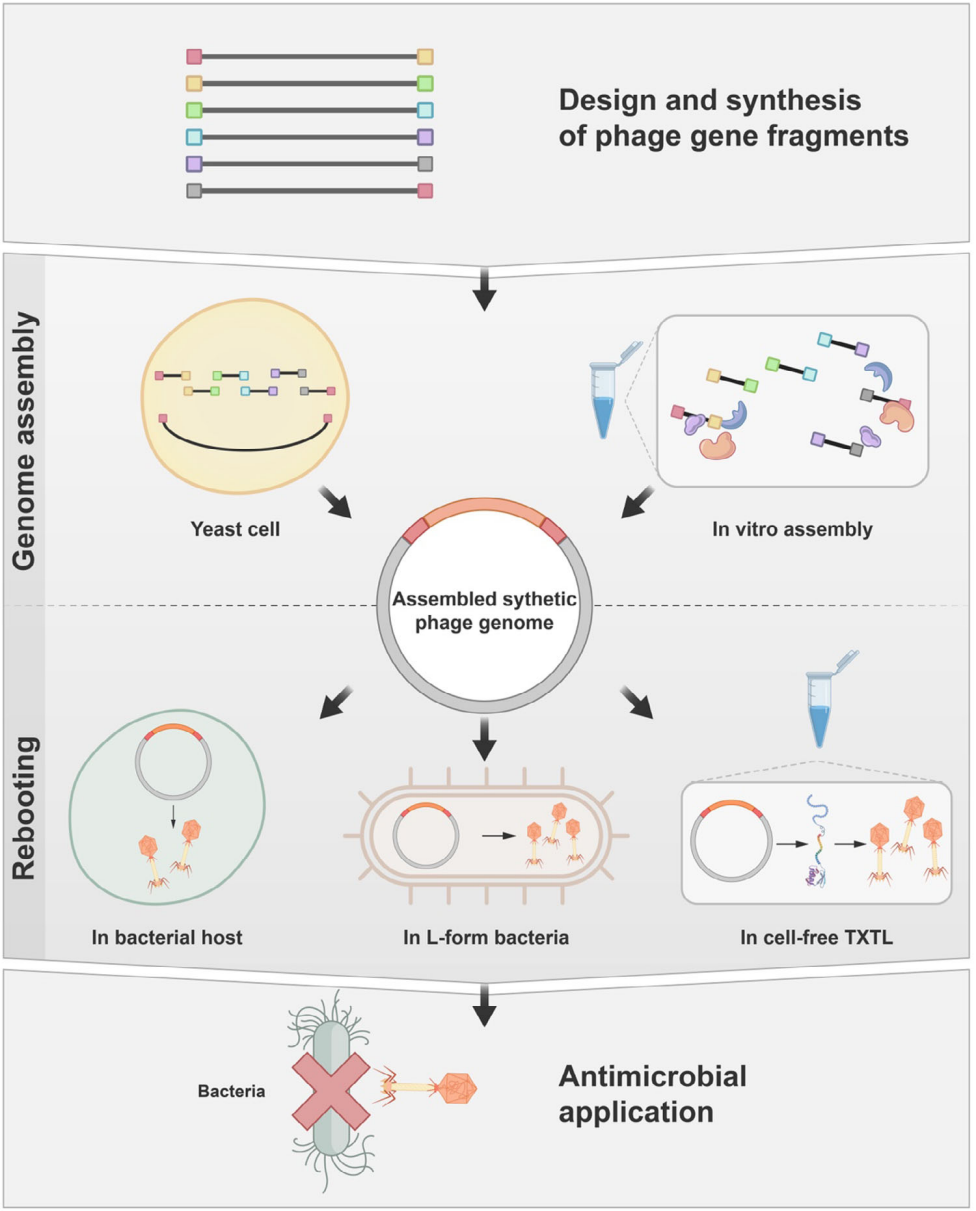
Phage synthesis engineering. Designed and synthesized gene fragments undergo homologous recombination within yeast cells or are assembled into a specific bacteriophage genome in vitro. Subsequently, the assembled synthetic phage genome can be rebooted within host cells, L‐form bacteria, or by utilizing cell‐free TXTL (transcription–translation) technology. Ultimately, the specific bacteriophage generated is utilized for antibacterial therapy.

Various strategies have been employed to enhance phage recombination efficiency. However, the modification of bacterial hosts still requires multiple iterations of editing and extensive screening efforts. This necessitates substantial investments of manpower, resources, and time for phage engineering. Additionally, it presents challenges such as a high number of procedural steps and a narrow margin for error. These factors somewhat limit the utility of bacteriophages in antimicrobial therapy, prompting the exploration of a more universally applicable and efficient technology. A novel approach involves synthesizing bacteriophages genomes to construct bacteriophages (Figure 2).

The synthesis of bacteriophages begins with genome assembly, a process initiated by amplifying gene segments through polymerase chain reaction (PCR). Subsequently, homologous recombination with yeast artificial chromosomes (YACs) and relevant bacteriophage gene segments can be utilized [57]. Currently, the most commonly utilized methods for in vitro synthesis of bacteriophage genomes are Gibson assembly [58] and Golden Gate [59] assembly. Audo et al. adapted this approach to modify bacteriophage genomes, thereby altering the host range of bacteriophages and achieving effective eradication of new target bacteria [57]. Another study documented phage assembly in yeast with minimal genes, leading to clearance of *P. aeruginosa* in the *Galleria mellonella* infection model [60].

Rebooting represents a pivotal stage in bacteriophage synthesis, entailing the transcription and translation of assembled bacteriophage segments, followed by their assembly into functional bacteriophages. However, conventional rebooting processes still rely on host cell transformation [58, 59, 60], which may not suffice for efficient transformation or the insertion of larger gene segments. Considering their ability to uptake larger fragments, L‐form bacteria, characterized as cell‐wall‐deficient, mononucleate variant of *Listeria*, emerge as a novel rebooting approach [61]. Kilcher et al. successfully utilized L‐form bacteria to activate large viral genomes (>130 kb), ultimately yielding viral particles [62]. Although currently primarily applied to Gram‐positive bacteria [63], this method offers opportunities for rebooting a broader range of bacteriophage genomes.

The synthesis and rebooting of bacteriophages in a cell‐free environment represent another crucial technique, overcoming limitations posed by genome size in bacteriophage engineering. This approach circumvents the need for intervention by live cells, facilitating rapid personalized production [64]. One commonly used cell‐free method is the TXTL system, which utilizes cytoplasmic extracts to initiate extracellular transcription and translation processes, with specific factors added as required [27]. Functional bacteriophages targeting Gram‐negative bacteria and *M. tuberculosis* have been successfully designed and synthesized by using this technology, which can effectively treat a pyogenic mouse model of bacterial infection [65]. Furthermore, the incorporation of this technique with CRISPR interference technology has increased the yield of in vitro synthesized T7 bacteriophages tenfold [66], laying the foundation for efficient and rapid production in the future bacteriophage therapy industry. Although cell‐free synthesis technology is still in its nascent stages and requires further exploration, it holds immense promise. Recent reports demonstrate the potential of this technology for achieving more complex gene additions, deletions, and mutations [67], providing strong support for the diverse applications of bacteriophage therapy in the future.

## 3 Engineering Microbes

The ongoing advancements in microbiomics have gradually unveiled the mechanisms underlying interactions and competition among microbes. Natural factors play a crucial role in maintaining relative stability within and between microbial populations, exerting dynamic control through positive activation or negative inhibition [68]. In the human gut, for example, the gut microbiota often produces substances like bacteriocins and organic acids, serving to inhibit the growth of other bacteria, particularly pathogenic invaders [69]. Consequently, these specifically secreted molecules can be harnessed to impede the activity of pathogens, offering a new avenue for exploring novel antimicrobial therapeutic approaches.

The utilization of engineering approaches in synthetic biology enables the design of microbes with specific functionalities, enabling them to produce antibacterial molecules targeting particular and precisely inactivating pathogens. Among antibacterial agents, bacteriocins stand out as the most prevalent type. They comprise a diverse group of antibacterial peptides or protease produced by bacteria and archaea, often displaying activity against species related to the producing bacterium. As mentioned previously, bacteriocins result from evolutionary processes and serve as a mechanism of specific immunity. In many cases, significant bactericidal efficacy can be achieved with relatively low concentrations [68]. *E. coli* Nissle 1917 serves as a commonly employed chassis bacterium [70], possessing inherent antibacterial mechanisms and immunomodulatory capabilities. Importantly, it has been demonstrated to be non‐lethal to the host, ensuring reliable safety [71]. Moreover, a comprehensive understanding of *E. coli* transcription and translation facilitates specific engineering modifications of Nissle 1917 [72]. Consequently, within the realm of antibacterial applications utilizing synthetic biology, Nissle 1917 is frequently targeted for engineering purposes.

**FIGURE 3:**
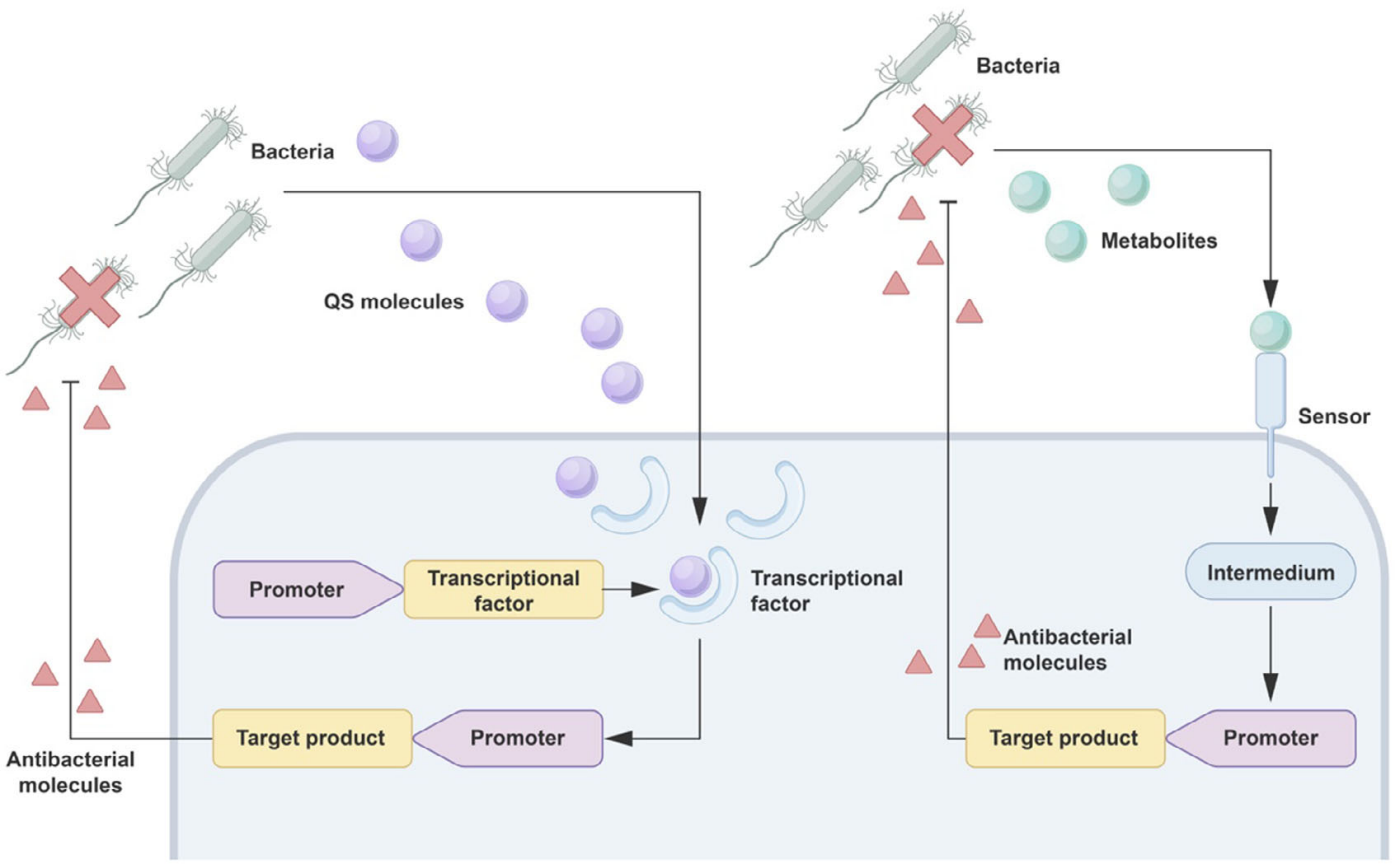
Sense‐killed system in engineering microbes. Engineered and modified microbes are designed to produce receptors or transcriptional factors that detect bacterial quorum‐sensing (QS) molecules (purple) or metabolites (green). Upon detection, the system triggers the production of molecules that are lethal to the bacteria.

Engineered microbes are also designed to sense specific metabolites or quorum sensing (QS) molecules produced by pathogens. This designed process involves modifying the cellular metabolic system to be responsive to these specific metabolites, thereby triggering downstream pathways and generating corresponding antibacterial molecules. This designed “sense‐killed” system [73] (Figure 3) represents a novel composite strategy for effective antibacterial action, offering new possibilities for future antibacterial therapy. Here, we mainly review experiments conducted using this strategy against common human pathogens (Table 2).

**TABLE 2:** Examples of engineered microbes for antibacterial therapy.

Target	Engineered microbes	Mechanism	Result
*P. aeruginosa*	*E. coli*	S5 and E7 proteins are released upon detection of AHL	*P. aeruginosa* was significantly reduced in vitro.
	*E. coli* Nissle 1917	In response to AHL, antimicrobial peptide microcin S and nuclease DNaseI are released.	The amount of *P. aeruginosa* was significantly reduced in vitro.
	*E. coli* Nissle 1917	The DspB biofilm enzyme is released on the basis of the above.	Bacterial biofilms were significantly reduced in mouse models.
	*E. coli* Nissle 1917	The antimicrobial peptide PA2‐GNU7 is released after the detection of AHL.	In mouse models, *P. aeruginosa* in the colon was significantly reduced.
*V. cholerae*	*E. coli* Nissle 1917	Stable expression of CAI‐1 and AI‐2 decreased virulence gene expression and bacterial colonization.	In mouse models, the toxin was reduced by 80% after 8 h and *V. cholerae* was reduced by 69% after 40 h.
	*E. coli*	After CAI‐1 was detected, the artilysins YebF‐Art‐085 is produced.	It can significantly inhibit the growth of *V. cholerae* and kill it effectively.
*Salmonella*	*E. coli* Nissle 1917	Engineered probiotics express antimicrobial peptides, Microcin J25.	In the Turkey model, *salmonella* was reduced by 97 percent.
	*E. coli* Nissle 1917	When a secondary metabolite of salmonella tetrathionate is detected, microcin H47 is produced.	Significantly inhibit the growth of *salmonella*.
*S. aureus*	*L. reuteri*	*L. reuteri* was designed to be able to detect AIP‐I for *S. aureus*.	Ability to detect AIP‐I levels in the nanomolar to micromolar range.
	*L. plantarum*WCSF	Lysostaphin is secreted after AIP is detected.	The inhibition of the *S. aureus* biofilm reached a level of 85%.

*Note: E. coli, Escherichia coli; L. reuteri, Lactobacillus reuteri; L. plantarum, Lactobacillus plantarum; P. aeruginosa, Pseudomonas aeruginosa; V. cholerae, Vibrio cholerae; S. aureus, Staphylococcus aureus*.

### 3.1 Targeted *Pseudomonas Aeruginosa*

*P. aeruginosa* is a prevalent gram‐negative pathogenic bacterium and a member of the ESKAPE group, comprising some of the most lethal bacterial pathogens. In clinical contexts, *P. aeruginosa* is frequently associated with ventilator‐associated pneumonia, a particularly life‐threatening condition for individuals with cystic fibrosis and those who are immunocompromised. Additionally, it can lead to sepsis, as well as infections of the skin and soft tissues [74].

The biosynthesis in *P. aeruginosa* is governed by QS, where *N*‐acyl homoserine lactone (AHL) serves as a crucial signaling molecule. When these signaling molecules reach a specific concentration, they activate regulatory factors, ultimately influencing gene expression. AHL plays a crucial role in various processes in *P. aeruginosa*, including biofilm formation, the production of secondary metabolites, the expression of virulence factors, and the development of resistance mechanisms [75]. Due to its pivotal role in bacterial population behavior and pathogenicity, targeting AHL sensing emerges as a promising strategy. In *E. coli*, this induction is achieved, for instance, by modifying *E. coli* to produce the transcription factor LasR. Upon sensing of the AHL, LasR binds to AHL, activates the luxR promoter, and induces the expression of lytic protein E7 and bacteriocin S5. When the concentration of E7 reaches a level sufficient to penetrate *E. coli*, S5 is released, eventually leading to *P. aeruginosa* death [73].

Hwang et al. further explored this approach by modifying *E. coli* to release the antibacterial peptide bacteriocin microcin S and the nuclease DNaseI in response to AHL, achieving bactericidal effects. Additionally, engineered *E. coli* can regulate the expression of CheZ under QS. In case of an imbalance with CheY, it migrates toward the target strain [76]. Subsequently, the team refined their approach by modifying the probiotic *E. coli* Nissle 1917 and incorporating dispersin B (DspB), an enzyme targeting biofilms. This optimization yielded positive results in both nematode and mouse models [77]. In a recent report, *E. coli* Nissle 1917 was similarly engineered, utilizing AHL as a responsive molecule. Upon AHL detection, the engineered *E. coli* produces an antibacterial peptide named PA2‐GNU7, effectively inhibiting the growth of *P. aeruginosa* [78].

### 3.2 Targeted *Vibrio Cholerae*

*V. cholerae*, a gram‐negative bacterium, is responsible for cholera, a severe gastrointestinal disease renowned for its high infectivity. Annually, cholera causes millions of infections and over a hundred thousand deaths globally [79]. The primary pathogenic mechanisms involve *V. cholerae* colonizing the small intestine and secreting cholera toxin. This exotoxin binds to GM1 ganglioside receptors on intestinal cells, continually activating adenylate cyclase linked to G proteins. This activation leads to an elevation in cAMP levels in epithelial cells of the small intestine, triggering a massive efflux of sodium ions and water. Ultimately, this process results in severe profuse diarrhea, vomiting, profound dehydration, and electrolyte imbalance [80].

Cholera toxin production is regulated by QS molecules. When the cell population reaches a specific concentration, it activates toxin expression. The QS system for cholera toxin primarily involves two sets of QS molecules, CAI‐1 and AI‐2. High concentrations of these inducers suppress the expression of virulence factors [81]. Consequently, modifying the engineered *E. coli* Nissle 1917 strain to express CAI‐1 and AI‐2 becomes a feasible strategy for customizing *V. cholerae* inhibition and toxin expression. A study revealed that this approach significantly reduced toxin production and mortality in a mouse model [82]. Similarly, a responsive template for sensing QS molecules can be designed, leading to the secretion of antibacterial molecules. Notably, genetically engineered *E. coli*, capable of detecting CAI‐1, a pivotal QS molecule in *V. cholerae*, produces the lytic protein YebF‐Art‐085. Upon release, this protein exerts lethal effects on *V. cholerae*, demonstrating a targeted antibacterial response [83].

### 3.3 Targeted *Salmonella*

*Salmonella*, a frequently encountered pathogenic bacterium in intestinal infections, is a causative agent of typhoid fever, particularly associated with a specific serotype of *Salmonella*. In the case of nontyphoidal Salmonella serotypes, the pathogen is often zoonotic, capable of cross‐species transmission between humans and animals, posing a health threat to both human and livestock populations [84]. Especially in the sub‐Saharan regions of Africa, nontyphoidal *Salmonella* infections are prevalent among febrile patients, with the pathogen frequently isolated from the blood, leading to severe infections. Additionally, the emergence of drug‐resistant strains, such as the ST313 strain has been noted. According to reports, in 2012, the mortality rate for diseases caused by nontyphoidal *Salmonella* infections reached 20–25% [85].

Forkus et al. conducted engineering on the *E. coli* Nissle 1917 strain to express the antibacterial peptide microcin J25. This modification resulted in a reduction of approximately 97% in *Salmonella* carriage in an infected turkey model [86]. Detecting the presence of *Salmonella* by engineered bacteria is crucial. Tetrathionate, a metabolic byproduct formed during inflammatory processes in the intestine through the reaction of reactive oxygen species with endogenous thiosulfate, serves as a key indicator. Concurrently, *Salmonella* utilizes thiosulfates to create a novel respiratory electron acceptor, giving it a distinct growth advantage compared to other microbial communities within the intestinal tract [87].

An illustrative example involves the engineering and editing of *E. coli* Nissle 1917 using tetrathionate as a responsive molecule. This modification enables the engineered strain to detect tetrathionate, subsequently inducing the expression of microcin H47. Microcin H47 has demonstrated effectiveness in inhibiting the growth of *Salmonella*, providing a targeted response to *Salmonella* infections [88].

### 3.4 Targeted Staphylococcus Aureus

*S. aureus*, a gram‐positive coccus and a member of the ESKAPE group, is a frequent causation in severe infections. It commonly invades the host's bloodstream, respiratory and gastrointestinal tracts, resulting in abscesses, pneumonia, endocarditis, osteomyelitis, sepsis, or other serious infectious diseases. Notably, it ranks among the most common causes of nosocomial infections in hospitals [89]. According to a 2017 report, the United States witnesses hundreds of thousands of cases of *S. aureus* bloodstream infections annually, leading to approximately 20,000 deaths. Of particular concern is the methicillin‐resistant *S. aureus* (MRSA) strain, which exhibits resistance to nearly all penicillins and most β‐lactam antibiotics, exacerbating mortality and morbidity rates [90].

Research indicates that *E. coli* Nissle 1917 can secrete a protease called DegP, capable of inhibiting biofilm formation. This inhibition effectively impedes the growth of *S. aureus* and *Staphylococcus epidermidis* [91]. A promising strategy involves further engineering bacteria to produce the DegP protease through thoughtful design. Moreover, the identification of suitable QS molecules plays a crucial role in constructing responsive modules in synthetic biology. Autoinducer peptide‐I (AIP‐1), produced by *S. aureus*, serves as a critical QS molecule in the pathogenic processes of this bacterium. This molecule operates within the Agr QS system, influencing biofilm formation and the regulation of virulence gene expression [92]. Leveraging these insights, *Lactobacillus reuteri* has been engineered to undergo Agr QS in response to AIP‐I from *S. aureus*. This biosensor is capable of detecting nanomolar concentrations of AIP‐I [93]. Recently, an approach was reported involving the modification of the plant‐associated bacterium *Lactobacillus plantarum* WCSF I to respond to AIP produced by the Agr QS system. This modification leads to the production of the bactericidal protein lysostaphin, effectively eradicating extracellular *S. aureus* in vitro [94].

Indeed, besides employing “sense‐killed” systems, engineered bacteria utilize alternative strategies to achieve antibacterial therapeutic goals. One such strategy involves immunological approaches. In addressing the challenge of combating drug‐resistant *C. difficile* infections, monoclonal antibody therapy is commonly employed. However, antibody injection is not only costly but also associated with low efficacy rates. Engineered *Saccharomyces boulardii*, capable of secreting a single tetra‐specific antibody, has been developed. This fusion antibody efficiently clears the pathogen in a *C. difficile*‐infected mouse model [95]. Additionally, another strategy involves utilizing and modifying other bacteria infecting the target organ to eliminate the target pathogen. For respiratory and pulmonary diseases caused by *P. aeruginosa* infections, a genetically modified attenuated strain of *Mycoplasma pneumoniae* (*M. pneumoniae*) M129 has been employed. After carrying the gene encoding biofilm hydrolases PelAh and PslG, as well as the bacteriocin S5, engineered *M. pneumoniae* demonstrated high efficacy in treating acute *P. aeruginosa* lung infections in a mouse model [96].

## 4 Engineering Mammalian Cells

In August 2017, the U.S. Food and Drug Administration (FDA) granted official approval for Novartis's therapeutic product, Kymriah (tisagenlecleucel), marking a significant milestone in cellular engineering. Kymriah, a chimeric antigen receptor T‐cell (CAR‐T) therapy, revolutionized the treatment of B‐cell acute lymphoblastic leukemia [15]. This approval paved the way for various CAR‐T therapies, heightening awareness of the clinical potential of cell therapy. Traditionally, scientific focus on cellular engineering was primarily directed towards bacteria. Bacteria‐based synthetic biology found application in diagnostics, such as detecting changes in serum zinc concentration [97] through in vitro analysis of biological samples like blood and urine, as well as in vivo applications for cancer [98] and inflammation [99] detection. Therapeutic potential in antibacterial treatment and specific cancer therapies was also explored [100].

**FIGURE 4:**
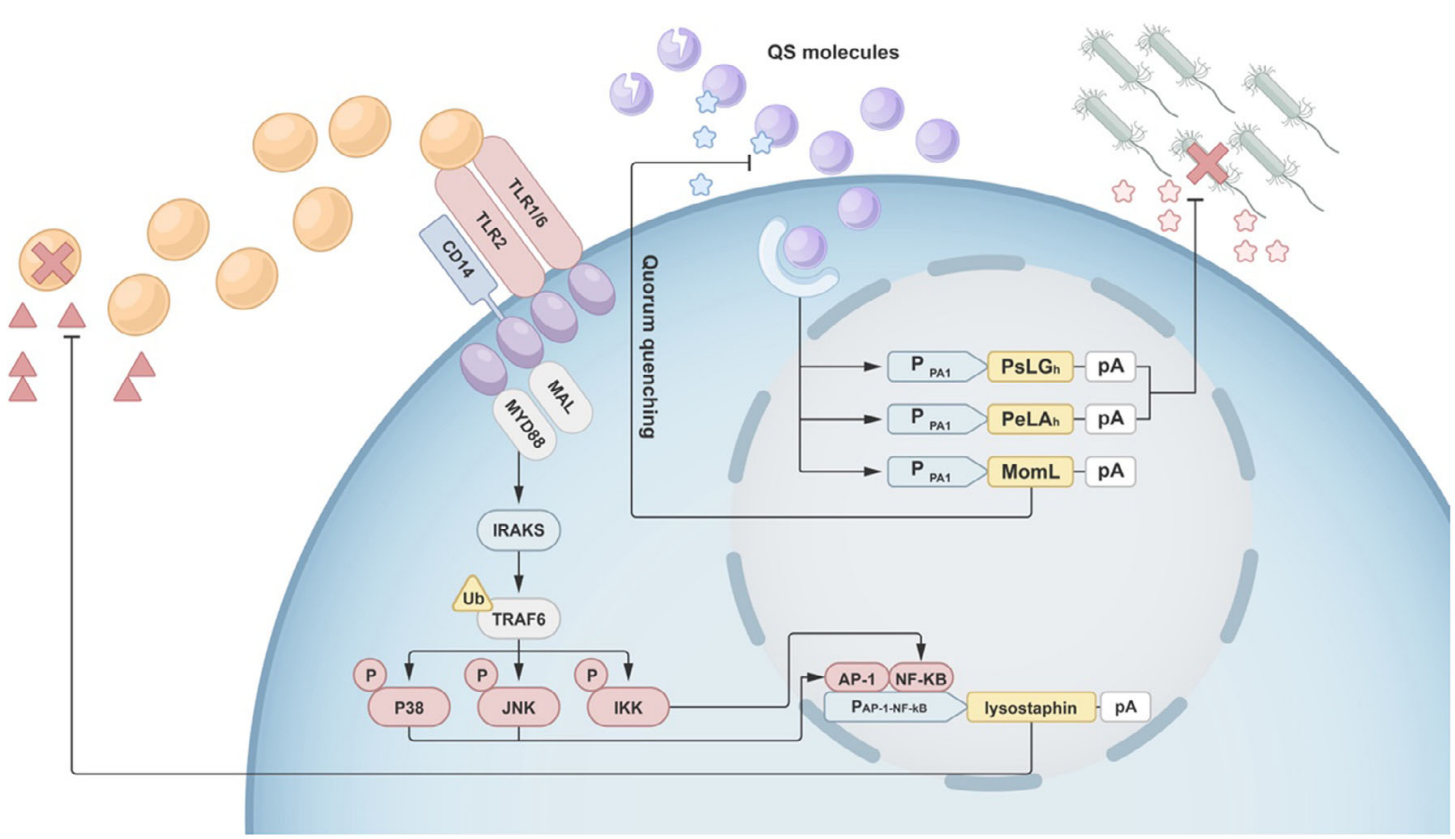
Antibacterial mechanisms in engineered mammalian cells. Engineering mammalian cells to produce a biological sensor involves the detection of bacterial quorum‐sensing (QS) molecules. Additionally, toll‐like receptors can be utilized to recognize bacterial pathogen‐associated molecular patterns (PAMPs). Upon recognition, the cells secrete molecules that are directly lethal to bacteria. Besides, engineering mammalian cells may also produce substances that control QS molecules, leading to the quorum‐quenching and collective suppression of bacterial activity.

However, bacterial‐based therapies face challenges. 1) Engineering bacterial therapies require a high concentration of bacteria in the bloodstream, potentially posing safety concerns that are difficult to rule out [101]. 2) Engineering bacteria may undergo mutations over time, leading to a decrease in therapeutic efficacy and potential adverse reactions [101]. 3) Engineering bacteria may be influenced by genetic material from other microbes, resulting in situations such as gene drift [102]. It is possible to enhance safety of engineered bacteria through measures like knocking out toxic genes [103] and incorporating suicide genes [104], but these approaches suffer from drawbacks such as increased experimental steps and cycles. Consequently, the use of mammalian cells in synthetic biology designs emerges as a promising solution to circumvent these challenges. Recent reports highlight the versatility of engineering mammalian cells in diagnostics and therapeutics [105]. For instance, engineering HEK293 cells enables the production of melanin when the concentration of calcium ions in the blood exceeds a certain threshold, allowing for the detection of hypercalcemia [106]. Engineering mammalian cells also holds therapeutic potential, extending beyond CAR‐T therapy to applications such as using HEK293 cells for treating psoriasis in cattle [107] and diabetes [108]. While there are relatively few reports on antibacterial treatment using engineered mammalian cells, their significant potential is evident in ongoing clinical development [109, 110, 111] (Figure 4).

### 4.1 QS‐Based Therapy

QS is a crucial microbial physiological behavior that relies on signaling molecules for information transfer, allowing bacteria to assess population density and respond to environmental changes. This process triggers the expression of specific genes, influencing essential bacterial physiological activities such as colonization, toxin production, and biofilm formation [112]. In the previous section, we discussed how engineered microbes can utilize QS as signaling molecules to activate specific pathways, leading to the secretion of substances for bactericidal purposes.

Similar strategies can be employed in engineering mammalian cells. Researchers engineered HEK‐293 cells to express a self‐inducing sensor (PAiS), enabling the cells to sensitively detect the self‐inducer PAI‐1 secreted by *P. aeruginosa*. Upon detection, downstream pathways are activated, resulting in the expression of glycoside hydrolase PslGh/PelAh, an enzyme capable of degrading the sugar moiety of *P. aeruginosa* biofilm. Furthermore, this engineering system can induce the expression of AHL‐lactonase MomL, which degrades AHL molecules [109]. Notably, when engineering cells detect bacteria, they not only secrete toxic molecules targeting the bacteria but also release an enzyme that degrades the QS molecule AHL. By utilizing QS molecules to control bacterial behavior, this approach reduces bacterial activity and impedes biofilm formation without directly killing the bacterial cells. This represents a novel strategy to achieve antibacterial objectives, referred to as “Quorum‐Quenching” in the aforementioned report. In another study, a similar strategy was employed to modulate key QS molecules for antibacterial purposes, specifically focusing on autoinducer‐2 (AI‐2), a crucial QS molecule influencing the quantity and gene expression of various microbial populations [113]. The team utilized a formyl‐peptide sensor (FPS) as a responsive module, sensitively detecting the formyl peptides secreted by microbes. This led to the generation of AI‐2, effectively controlling pathogenic behaviors in *Vibrio harveyi* and inhibiting biofilm formation by *Candida albicans* [110].

### 4.2 Immunological‐Based Therapy

The application of engineering animal cells predominantly relies on immune recognition‐based sensors. An illustrative example is the engineering of CAR‐T cells, where host T cells are modified to express antigen receptors specific to cancer cells, facilitating the targeted destruction of cancerous cells [114]. Similarly, in the context of employing mammalian cells for antibacterial therapy, responsive modules are based on immune‐related sensors. A crucial category of proteins in the innate immune system is Toll‐like receptors (TLRs). When microbes invade, the conserved sequences (PAMPs, pathogen‐associated molecular patterns) within them can be recognized by TLRs, activating the immune system to eliminate the pathogens [115]. Exploiting this mechanism, Liu and colleagues designed cells containing TLR2 and TLR1/TLR6, which can sensitively detect *S. aureus* invasion and release lysostaphin. This approach effectively eradicates *S. aureus* with a 100% cure rate and diminishes MRSA biofilm formation by 91% [111].

## 5 Conclusion and Discussion

In the past decade, synthetic biology has made significant advancements due to the development of gene‐editing technologies [10]. This progress has been translated into various fields including medicine, chemistry, environment, agriculture, food etc. In the medical field specifically, there have been extensive experiments and practical applications involving the engineering and synthesis of life forms for disease diagnosis and treatment [10]. Currently, as antibiotic‐resistant bacteria pose a threat, there is an emerging trend to use synthetic biology methods as a novel therapeutic strategy to replace traditional antibiotics for treating bacterial infections. This summary provides an overview of techniques used for modifying bacteriophages, microbes, and mammalian cells with potential implications for antibacterial treatments.

Bacteriophages have been recognized as one of the earliest antibacterial agents, used extensively before antibiotics became widely available. However, natural bacteriophages often exhibit limited treatment effectiveness and can only target specific hosts. By genetically modifying bacteriophages, researchers have achieved significant success, especially when combined with antibiotics [116]. Despite greater prospects compared to traditional antibacterial methods, engineered bacteriophage therapy still faces certain challenges related to biosafety. Additionally, the absence of an established regulatory framework for bacteriophage therapy has hindered its progress [117].

The engineering of microbes is an alternative approach, specifically in modifying probiotics. Engineered probiotics are stable and specific, cost‐effective, and have been used for diagnosing and treating certain diseases [118]. In antibacterial therapy, researchers have utilized the unique bacterial QS to create a sense‐killed system, where engineering microbes act as biological switch when sensing pathogens. Such a biological component can autonomously detect the presence of pathogens, enable the precise and controlled targeting and destruction of pathogenic bacteria. However, biosafety remains a significant concern for this method, similar to bacteriophages, which required considerable scrutiny.

Compared to engineering bacteriophages and microbes, engineering mammalian cell‐based therapies exhibit superior biosafety and enhanced compatibility with the human body. Particularly in the clinical application of CAR‐T therapy, cell therapy has garnered extensive attention. In antibacterial treatment, engineering mammalian cells also emerge as an optimal solution. However, they encounter challenges associated with economic considerations and costs. For instance, CAR‐T therapy often entails substantial expenses, reaching hundreds of thousands of dollars. This factor contributes to the relatively limited research on cell therapy in the antibacterial field compared to oncology, where the economic benefits of anticancer treatments outweigh those associated with antibacterial interventions.

In summary, synthetic biology holds tremendous potential for applications, and researchers' relentless exploration of novel antibacterial strategies instills hope in combating drug‐resistant bacteria. Despite the myriad of challenges we currently face there is reason to believe that with the continual advancement of technology and the deepening of theoretical understanding, an increasing amount of research will translate into clinical applications. Furthermore, with the rise of artificial intelligence, there will be increasing integration between large‐scale models and synthetic biology. This integration holds the promise of harnessing diverse biological components to craft more systematic genetic circuits. It also entails utilizing artificial intelligence to forecast the efficacy and efficiency of current genetic circuits, and even to devise and pinpoint novel regulatory units and networks [119]. In the future, we can expect the emergence of more efficient, user‐friendly, and cost‐effective products in this field within our review.

## Conflicts of Interest

The authors declare no conflicts of interest.
